# Ultrasonication-assisted synthesis of CsPbBr_3_ and Cs_4_PbBr_6_ perovskite nanocrystals and their reversible transformation

**DOI:** 10.3762/bjnano.10.66

**Published:** 2019-03-06

**Authors:** Longshi Rao, Xinrui Ding, Xuewei Du, Guanwei Liang, Yong Tang, Kairui Tang, Jin Z Zhang

**Affiliations:** 1 Engineering Research Centre of Green Manufacturing for Energy-Saving and New-Energy Technology, School of Mechanical and Automotive Engineering, South China University of Technology, Guangzhou 510640, China; 2 Department of Chemistry and Biochemistry, University of California, Santa Cruz, CA 95064, USA; 3 Mechanical Engineering, Pennsylvania State University, Harrisburg, PA 17057, USA

**Keywords:** CsPbBr_3_ PNCs, Cs_4_PbBr_6_ PNCs, polar-solvent-free, reversible transformation, ultrasonication

## Abstract

We demonstrate an ultrasonication-assisted synthesis without polar solvent of CsPbBr_3_ and Cs_4_PbBr_6_ perovskite nanocrystals (PNCs) and their reversible transformation. The as-prepared CsPbBr_3_ PNCs and Cs_4_PbBr_6_ PNCs exhibit different optical properties that depend on their morphology, size, and structure. The photoluminescence (PL) emission and quantum yield (QY) of the CsPbBr_3_ PNCs can be tuned by changing the ultrasound power, radiation time, and the height of the vibrating spear. The optimized CsPbBr_3_ PNCs show a good stability and high PL QY of up to 85%. In addition, the phase transformation between CsPbBr_3_ PNCs and Cs_4_PbBr_6_ PNCs can be obtained through varying the amount of oleylamine (OAm) and water. The mechanism of this transformation between the CsPbBr_3_ PNCs and Cs_4_PbBr_6_ PNCs and their morphology change are studied, involving ions equilibrium, anisotropic growth kinetics, and CsBr-stripping process.

## Introduction

Metal halide perovskite nanocrystals (PNCs) are promising candidates for application in the fields of light-emitting diodes (LEDs) [1–2], high-efficiency solar cells [3], low-threshold lasers [4], and photodetectors [5]. Compared to traditional semiconductors, colloidal PNCs demonstrate excellent properites, such as tunable photoluminescence (PL) throughout the visible spectrum, super high PL quantum yield (QY), low trap-state density, and narrow emission linewidth [6–8]. The crystal structure of CsPbX_3_ (X = Cl^−^, Br^−^, I^−^) PNCs consists of a 12-fold coordinated network created by [PbX_6_]^4−^ octahedra in which the Cs^+^ ions reside in the periphery of this network [9–10]. These PNCs are prone to structural instabilities and phase transformations involving ion migration and interface hydration [11]. However, this phase and structure versatility has become the great advantage of PNCs in their technical applications, especially in optoelectronics. Although the focus has been on the CsPbX_3_ structure, researchers start to turn their attention on synthesizing new perovskite materials, such as Cs_4_PbX_6_ PNCs. Under Cs-rich or Pb-poor synthesis conditions, zero-dimensional (0D) structures of Cs_4_PbX_6_ NCs can be achieved, demonstrating a crystalline structure with well-separated octahedra [PbBr_6_]^4−^ isolated by Cs^+^ ions [12–13]. This specific structure is expected to result in strong quantum confinement and electron–phonon interactions. This inspires researchers to further explore this structure. The key to this exploration is the development of various approaches to the synthesis of high-quality PNCs.

Since, in 2015, Kovalenko and co-workers synthesized CsPbX_3_ PNCs by using a hot-injection method, great successes in the controlled synthesis and application of the CsPbX_3_ PNCs have been achieved in a very short time [14]. To date, the most commonly adopted approach for synthesizing highly efficient PNCs are solution-based procedures, including hot injection, solvothermal synthesis, microreactor synthesis, and room-temperature (RT) ligand-mediated reprecipitation, in which shape and size are tuned through the control of temperature, reaction time, and composition of the precursors [15–17]. Chen et al. demonstrated a facile solvothermal method for preparing CsPbX_3_ PNCs with adjustable optical properties [18]. Additionally, Li's group reported a poly(lactic acid)-assisted anion-exchange method using a microreactor for tuning the emission spectra of CsPbX_3_ PNCs from green to near-ultraviolet, which might be applicable for mass production [19]. Besides, great efforts have been made to prepare PNCs through the chemical transformation of pre-synthesized PNCs [20–22]. For example, Wu et al. reported a CsX-stripping method that enabled the transformation of nonluminescent Cs_4_PbX_6_ PNCs to highly luminescent CsPbX_3_ PNCs through an interfacial reaction [20]. They focus on investigating the water-triggered transformation process between Cs_4_PbX_6_ PNCs and CsPbX_3_ PNCs in a different phase. Similar methods were applied to explore new perovskite materials such as Cs_4_PbBr_6_. Zhai et al. showed a simple solvothermal approach for synthesizing CsPbBr_3_ nanoplatelets and their phase transformation to Cs_4_PbBr_6_ PNCs [23]. Liu and co-workers also demonstrated that CsPbBr_3_ PNCs were successfully converted to Cs_4_PbBr_6_ PNCs through a “ligand-mediated transformation” method with the addition of oleylamine (OAm) [24]. Udayabhaskararao’s group showed the reversible transformation from CsPbX_3_ to Cs_4_PbX_6_ through the ratio of oleic acid (OA) to OAm in a Brønsted acid–base equilibrium [25]. Despite the progress made in obtaining PNCs, in general, inert conditions, high temperature and pre-synthesized precursors are required for hot injection. In addition, RT methods were mostly carried out by mixing a polar solvent with a large amount of nonpolar solvent. Since PNCs are reported to be very sensitive to polar solvents, these methods result in the inevitable degradation of PNCs, especially for iodine-based PNCs [26–28]. Therefore, in order to obtain PNCs with high PL QY and stability, it is crucial to develop synthesis methods free of polar-solvents.

To date, some attempts have been made to synthesize PNCs without the use of polar solvents. Tong’s group demonstrated the single-step and polar-solvent-free synthesis of CsPbX_3_ PNCs with tunable halide ion composition and thickness through the direct ultrasonication of precursors [29]. Whereas this method has been reported for synthesizing PNCs without using polar solvents, it does not allow for a control over dimensionality and phase transformation. We recently reported a fast, low-cost, environmentally friendly, and polar-solvent-free strategy for synthesizing all-inorganic CsPbBr_3_ NCs with tunable shape and size [30]. During this process, we found that a great excess of OAm results in the formation of a derivative of CsPbBr_3_ NCs, i.e., Cs_4_PbBr_6_. However, the underlying transformation mechanism has not been fully understood. Following this, we set out here to expand this study to control the phase transformation. CsPbBr_3_ PNCs as precursor were obtained by modifying the approach initially presented by Tong, which was recently elaborated by our group [29–30]. We demonstrated in detail how, by tuning the ultrasound power and time, the PL emission of CsPbBr_3_ PNCs can be precisely controlled. Benefitting from this knowledge, here we attained CsPbBr_3_ PNCs with a high PL QY (ca. 85%) by optimizing the immersion height of the vibrating spear in the liquid. In addition, the phase transformation of CsPbBr_3_ PNCs to Cs_4_PbBr_6_ PNCs was achieved in this study by direct ultrasonication of solid powders or by adding OAm in the solution of pre-synthesized CsPbBr_3_ PNCs. Finally, inspired by the method proposed by Wu et al. [20], a successful structure conversion from Cs_4_PbBr_6_ PNCs to CsPbBr_3_ PNCs was obtained here by simply adding different amounts of water into pre-synthesized Cs_4_PbBr_6_ PNCs. The mechanism behind phase transformation and morphology change were investigated by using a combination of spectroscopy and microscopy techniques.

## Results and Discussion

### Characterization of CsPbBr_3_ PNCs

**Scheme 1:**
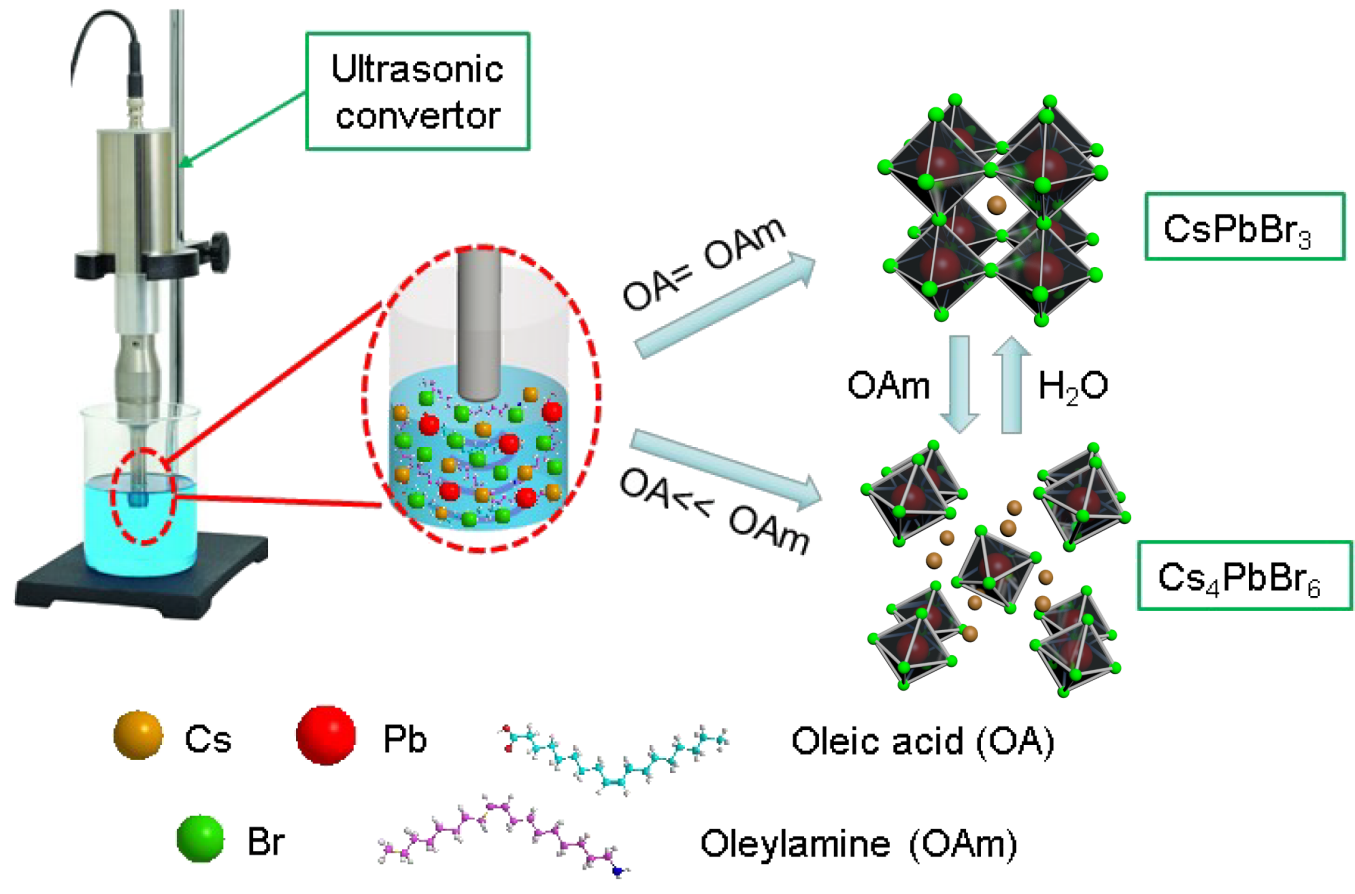
Illustration of synthesizing CsPbBr_3_ and Cs_4_PbBr_6_ PNCs without polar solvent using ultrasonication assistance, and their reversible transformation by adding OAm and water.

The typical procedure for synthesizing CsPbBr_3_ and Cs_4_PbBr_6_ PNCs and for reversibly transforming them is illustrated in Scheme 1. Cs_2_CO_3_ and PbBr_2_ were loaded into the liquid paraffin/OAm/OA solution. Then, the precursors were processed by tip-sonication and purified via centrifuging in the presence of methyl acetate as precipitation agent. Subsequently, the sediment was redispersed in toluene for further characterization. The reversible transformation between Cs_4_PbBr_6_ PNCs and CsPbBr_3_ PNCs was achieved by changing the amounts of OAm and water. Detailed synthesis conditions are given in the Experimental section.

**Figure 1:**
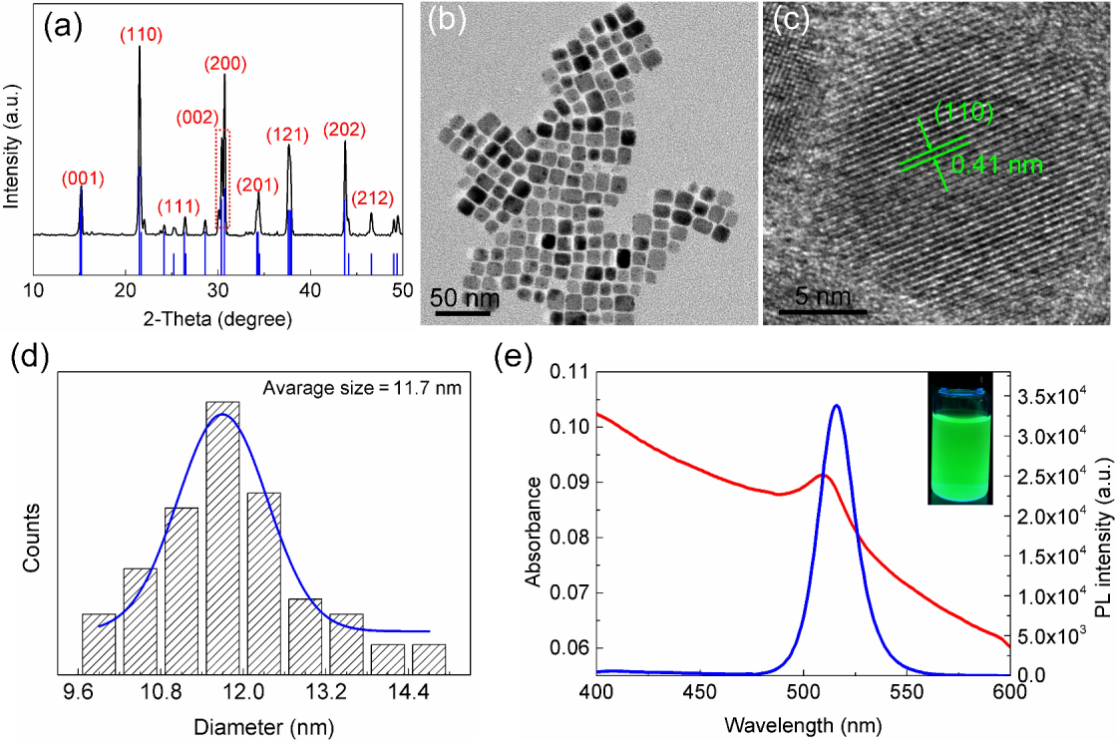
Characterization of the CsPbBr_3_ PNCs prepared using ultrasonication assistance. (a) XRD patterns. Black line and blue line represent experimental data and standard reference, respectively. (b) TEM image. (c) HRTEM image. (d) Size distribution. (e) UV–vis absorption spectrum (red line) and PL emission spectrum (blue line). Inset is a photograph under 365 nm UV irradiation.

The crystal structure and morphology of the as-prepared samples were determined by XRD and TEM. As shown in Figure 1a, the diffraction pattern clearly indicates that orthorhombic CsPbBr_3_ PNCs (PDF card #18-0364) were formed. No other phases were observed, suggesting the high purity of the samples. The TEM image shown in Figure 1b demonstrates that the CsPbBr_3_ PNCs have a regular square morphology. HRTEM was further carried out to measure the lattice spacing of the product. Figure 1c shows a lattice spacing distance of ca. 0.41 nm for the CsPbBr_3_ PNCs. The size distribution shown in Figure 1d indicates that the well-dispersed CsPbBr_3_ PNCs have an average diameter of ca. 11.7 nm. To explore the optical properties of colloidal CsPbBr_3_ PNCs, UV–vis absorption spectra and PL emission spectra were recorded. As shown in Figure 1e, the first excitonic absorption peak was located at 510 nm and the strong PL emission band centered at 516 nm was observed with a narrow full width at half maximum (FWHM) of 18 nm, indicating a narrow polydispersity of the PNCs obtained by this method. The PL QY of the as-prepared CsPbBr_3_ PNCs measured to be ca. 85% (Rhodamin 101 as reference, PL QY is 100%) following a previously published report [31]. In addition, Supporting Information File 1, Figure S1 clearly demonstrates the improved photostability and chemical stability of CsPbBr_3_ PNCs.

### Effect of synthesis conditions

Our previous study has shown that ultrasound power and radiation time have a great influence on the optical properties of the CsPbBr_3_ PNCs [30]. In this study, we found that the immersion height of the vibrating spear in the solvent influences the product properties (the effect will be discussed later). We divided the height of liquid into five equal parts, i.e., from the bottom to the surface of the liquid, 1/5, 2/5, 3/5, 4/5, and 5/5.

**Figure 2:**
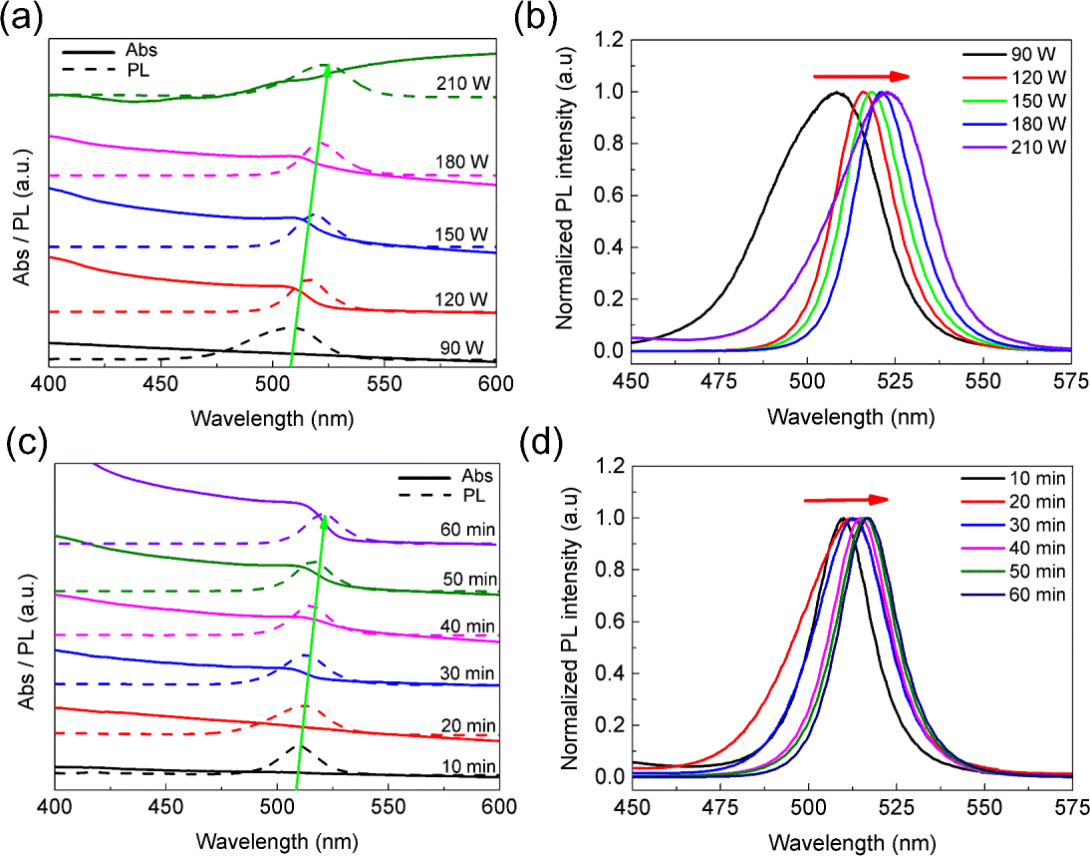
(a) UV–vis absorption spectra and PL emission spectra, and (b) normalized PL emission spectra of the CsPbBr_3_ PNCs that were synthesized at 90, 120, 150, 180, and 210 W of ultrasound power, respectively. (c) UV–vis absorption spectra and PL spectra, and (d) normalized PL emission spectra of CsPbBr_3_ PNCs that were synthesized at 10, 20, 30, 40, 50, and 60 min of radiation time.

We first investigated the effect of ultrasound power on the optical properties of CsPbBr_3_ PNCs. To avoid breaking the bottle, the immersion height of the vibrating spear and radiation time are 4/5 and 30 min, respectively. Figure 2a shows the change of UV–vis absorption spectra and PL spectra of the CsPbBr_3_ PNCs that were synthesized at 90, 120, 150, 180, and 210 W of ultrasound power, while keeping other synthesis conditions unchanged. If the ultrasound power is less than 90 W, there is no UV–vis absorption peak and a very weak PL intensity, implying that almost no PNCs formed. However, the first characteristic absorption peak changes to red slowly with an increase of ultrasound power, corresponding to the red-shift of the PL emission peak, which is similar to the findings we recently reported [30]. While higher ultrasound power supports faster dissolution, it has also a strong impact on the homogeneity of the PNCs. For example, when the ultrasound power is 210 W, the UV–vis absorption at long wavelengths is very high, indicating large crystals were formed with strong scattering. Therefore, it is necessary to choose the appropriate ultrasound power. The normalized PL emission peaks in Figure 2b shift from 505 to 523 nm, indicating that our approach can precisely modulate PL emission.

Furthermore, we studied the influence of radiation time on the CsPbBr_3_ PNCs. The immersion height of the vibrating spear and ultrasound power are 4/5 and 120 W, respectively. As shown in Figure 2c and Figure 2d, when the radiation time is increased, both the UV–vis absorption and PL spectra are red-shifted, we suggest that it is the size effect that is dominant over ionic bond strength in causing the spectral shift, which is different from the effects of radiation time that we observed recently [30]. This phenomenon indicates that the immersion height of the vibrating spear would affect PNCs properties.

**Figure 3:**
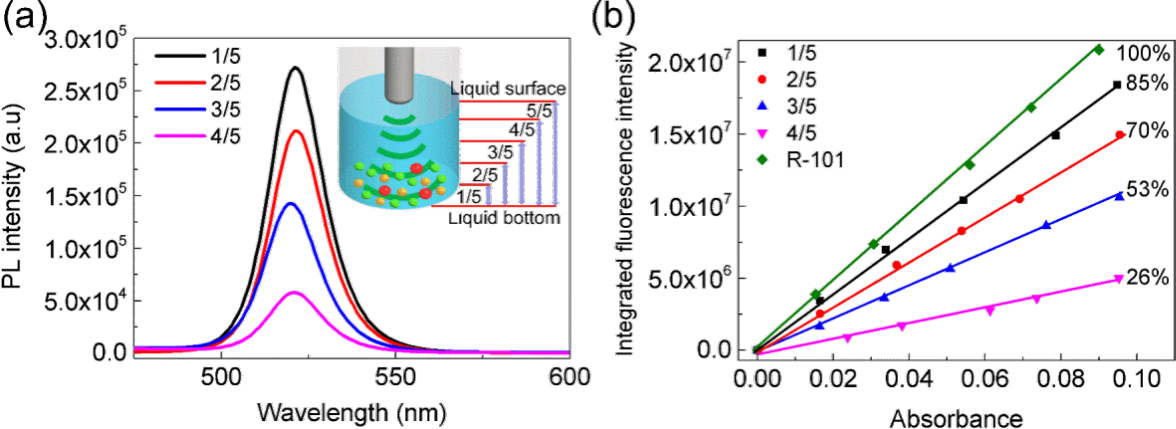
(a) PL emission spectra and (b) PL QY of the CsPbBr_3_ PNCs that were synthesized at 1/5, 2/5, 3/5, and 4/5 of total liquid height, respectively. 1/5, 2/5, 3/5, 4/5, and 5/5 in (a) inset is one of the five equal parts from liquid bottom to liquid surface. R-101 in (b) is rhodamine 101 (PL QY, 100%) as the standard sample to test PL QY of PNCs.

The effects of the immersion height of the vibrating spear in the liquid were also investigated. The total liquid height was divided into five equal parts as shown in the inset of Figure 3a. Figure 3a shows the PL intensity (UV–vis absorbance at 400 nm) of four samples that were synthesized by setting the immersion height of the vibrating spear to 1/5, 2/5, 3/5, and 4/5 of the total liquid height. As the immersion height increases, the corresponding PL intensity obviously decreases. In addition, the PL QY in Figure 3b further confirmed that the CsPbBr_3_ PNCs exhibit the best performance when the immersion height of the vibrating spear is set at 1/5 of the total liquid height. Ultrasonication results in a combination of thermal, vibrational, and acoustic cavitation, i.e., the formation, growth, and implosive collapse of bubbles in liquids [32–34]. In the center of these bubbles, extremely high temperatures of about 5000 K and high pressures of about 20 MPa were achieved by high-intensity ultrasound [32], enabling a quick decomposition of the particles. The lower the immersion height of vibrating spear, the higher temperature and pressure is achieved, which, as a result, benefits the formation of PNCs.

### Characterization of Cs_4_PbBr_6_ PNCs

**Figure 4:**
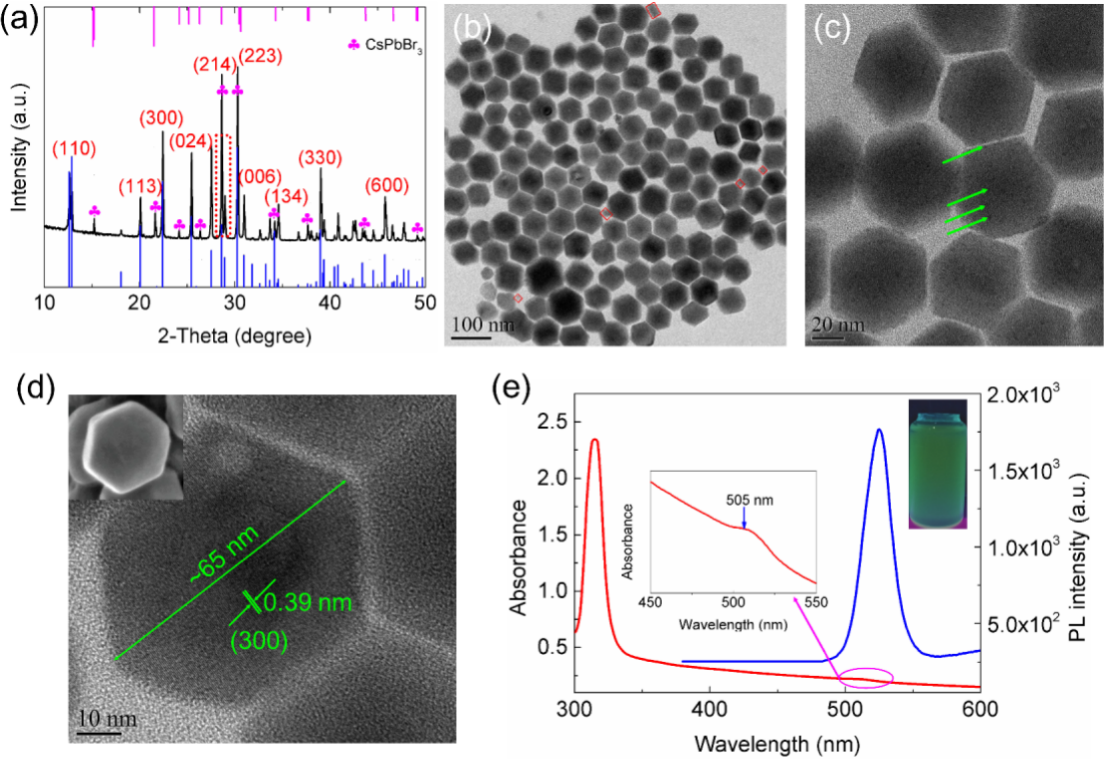
Characterization of the Cs_4_PbBr_6_ PNCs synthesized using ultrasonication. (a) XRD patterns. Black line represents experimental data; blue line (below) and pink line (upper) represent standard reference bulk CsPbBr_3_ and Cs_4_PbBr_6_, respectively. (b) TEM image, red squares represent CsPbBr_3_ PNCs. (c),(d) HRTEM images, green arrows in (c) represent metallic lead nanoparticles and inset in (d) is SEM image of Cs_4_PbBr_6_ PNCs, respectively. (e) UV–vis absorption spectra (red line) and PL emission spectra (blue line). Insets are high resolution absorption spectrum and representative digital photograph.

The current approach can be further used for controlling phase and structure transformations in the PNCs. The method introduced in this work enables the successful synthesis of rhombohedral Cs_4_PbBr_6_ PNCs via changing the amount of OAm. The amount of OAm was increased to 3.0 mL, while all other conditions were kept the same. The phase of the obtained product was characterized by XRD, as shown in Figure 4a. The XRD pattern with peaks at 2θ = 12.9, 20.1, 22.4, 25.6, 28.6 30.3, 30.9, 34.1, 39.3, and 45.7° correspond to diffractions from (110), (113), (300), (024), (214), (223), (006), (134), (330), and (600) crystal planes of rhombohedral Cs_4_PbBr_6_ (PDF card #73-2478) [13]. Meanwhile, weak peaks of CsPbBr_3_ were observed, indicating both CsPbBr_3_ and Cs_4_PbBr_6_ PNCs were formed during the process.

TEM was further performed to characterize the morphology of as-prepared PNCs. Figure 4b shows the formation of Cs_4_PbBr_6_ PNCs with hexagonal crystal structure and confirms the existence of square-shaped CsPbBr_3_ PNCs. Additionally, Figure 4c shows that the small black spots (green arrows) existing on the surface of the Cs_4_PbBr_6_ PNCs are metallic lead nanoparticles that have been reported before [23,35–36]. The HRTEM image shown in Figure 4d demonstrates an interplanar spacing of 0.39 nm, corresponding to the (300) crystal plane of bulk Cs_4_PbBr_6_, which is also consistent with the PDF card #73-2478. The size of the Cs_4_PbBr_6_ PNCs is defined here as the longest distance between hexagonal corners, which is ca. 65 nm for the example shown. Besides, the SEM image illustrates that Cs_4_PbBr_6_ particles are hexagonal prisms with a thickness of ca. 15 nm, as presented in Figure 4d inset and Supporting Information File 1, Figure S2.

The absorption spectrum of the Cs_4_PbBr_6_ PNCs is very different from that of the CsPbBr_3_ PNCs, as shown in Figure 4e. The first excitonic absorption has been shifted from 510 nm for CsPbBr_3_ PNCs to 315 nm for Cs_4_PbBr_6_ PNCs. This absorption feature is consistent with that of bulk Cs_4_PbBr_6_, which was proven to be the localized 6s_1/2_–6p_1/2_ transition within the isolated [PbBr_6_]^4−^ octahedra separated by Cs^+^ ions [24]. A weak characteristic UV–vis absorption and a PL emission peak for CsPbBr_3_ PNCs at 505 nm and 520 nm, respectively, further confirmed the existence CsPbBr_3_ PNCs.

In Cs_4_PbBr_6_ PNCs, typically a green emission arises either from defects or from impurities or from a combination of both [37–38]. Herein, the purified Cs_4_PbBr_6_ PNCs did not demonstrate PL emission over the whole visible spectrum due to their wide bandgap (*E*_g_(Cs_4_PbBr_6_) = 3.94 eV), while the observed weak PL emission results from a small portion of CsPbBr_3_ impurities in the Cs_4_PbBr_6_ PNCs (see Figure 3a–e). Since CsPbBr_3_ PNCs exhibit a high PL QY, the green PL emission is ascribed to minor CsPbBr_3_ impurities in the samples. This result coincides with previous works on Cs_4_PbBr_6_ PNCs that show a strong green emission at about 500 nm and confirmed that these green PL emissions originate from CsPbBr_3_ PNC impurities [10,39–40].

**Figure 5:**
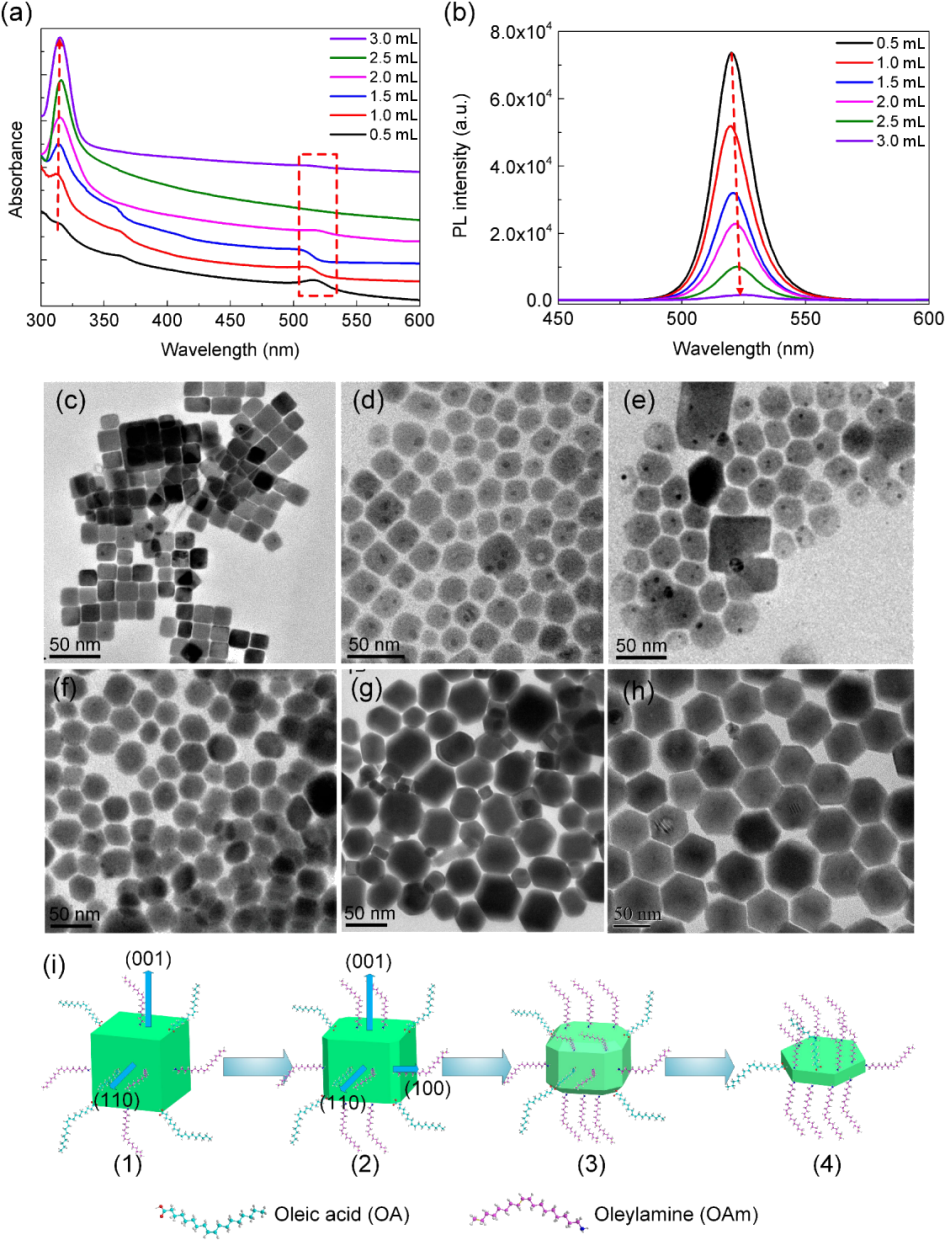
The change of (a) UV–vis absorption spectra, (b) PL spectra, and (c)–(h) TEM images of CsPbBr_3_ PNCs synthesized at 0.5, 1.0, 1.5, 2.0, 2.5, and 3.0 mL of OAm, respectively, while keeping OA unchanged. (i) Schematic illustration of the morphology change from CsPbBr_3_ PNCs to Cs_4_PbBr_6_ PNCs.

Furthermore, the effect of the amount of OAm on the phase transformation was investigated. As shown in Figure 5a, when the amount of OAm ranged from 0.5 to 3.0 mL, the first characteristic absorption peak (ca. 510 nm) and the PL emission intensity of CsPbBr_3_ PNCs slowly decrease, while new strong absorption features in the UV region (ca. 315 nm) emerge, which have been confirmed to result from the formation of Cs_4_PbBr_6_ PNCs [25]. When adding equal amounts of OAm and OA, there are no other peaks in the UV–vis absorption spectra except for that at ca. 510 nm. With increasing amount of OAm, the absorption intensity at ca. 510 nm decreases. Simultaneously, the absorption intensity at ca. 315 nm increases, while the PL intensity decreases (Figure 5b), and is blue-shifted followed by an increase in the FWHM of the PL peak. All these effects suggest the decomposition of the CsPbBr_3_ PNCs. Based on this process it can be concluded that the excess amount of OAm triggers the transformation between CsPbBr_3_ PNCs and Cs_4_PbBr_6_ PNCs.

The morphology change from CsPbBr_3_ PNCs to Cs_4_PbBr_6_ PNCs was further confirmed by using TEM. When the amount of OAm is between 0.5 and 1.5 mL, the morphology of PNCs gradually becomes irregular and some hexagonal shapes emerge (Figure 5c–e). As the amount of OAm increase to 3.0 mL, Cs_4_PbBr_6_ PNCs with homogeneous hexagonal shape can be achieved (Figure 5h). These results suggest that the growth kinetics of this process can be controlled by adding OAm, and the PNCs are prone to crystallize in the Cs-rich Cs_4_PbBr_6_ phase when OAm is present in excess. The transformation from CsPbBr_3_ to Cs_4_PbBr_6_ leads to a remarkable change in crystal structure and atomic composition. Udayabhaskararao et al. demonstrated that this transformation is driven by recrystallization induced by micelle formation or soft-ligand templating [25]. This mechanism, however, cannot explain the phase transformation from CsPbBr_3_ to Cs_4_PbBr_6_ in this work because there are no intermediate stages observed by TEM. Therefore, we suppose the transformation between the two phases involves ion equilibria. A large amount of OAm can form oleylammonium and dissolve PbBr_2_, resulting in the formation of lead oleate and oleylammonium bromide, thus driving the transformation [41]. An even larger amount of OAm can also dissolve CsPbBr_3_ PNCs and accelerate the transformation into Cs_4_PbBr_6_ PNCs. This process is related to Ostwald ripening that was found during the nucleation and growth of PNCs [42]. Therefore, the formation of Cs_4_PbBr_6_ PNCs is promoted by the capacity of the organic ligands to dissolve PbBr_2_ and by the dissociation of CsPbBr_3_ PNCs.

A series of TEM images (Figure 4c–h) clearly confirm that the morphology of these PNCs can be tuned easily by changing the amount of OAm in the precursor solution, while keeping the amount of OA unchanged. We attribute this morphology change to the crystal anisotropy induced by the growth kinetics. The capping ligands are preferentially attached to the PNCs facets, resulting in different growth rates on different crystal facets [43]. A schematic illustration of the morphology change between CsPbBr_3_ PNCs and Cs_4_PbBr_6_ PNCs is shown in Figure 5i. When equal amounts of OA and OAm are added, the reaction favors isotropic growth, since OA and OAm play a cooperative role (Figure 5i(1)). When more OAm is added, the long-chain OAm are more easily bound to the surface of the PNCs and restrict the perpendicular growth (001) [44]. Additionally, the growth rates for the side planes are different due to excess amount of OAm easily aggregated at the boundary of two adjacent planes [45], which possibly leads to the formation of (100) planes and the appearance of diamond-like product, as demonstrated in Figure 5i(2,3). This inhibiting effect is distinct when OAm is added in large excess, yielding a hexagonal structure with sharp edges, as shown in Figure 5i(4).

### Reversible transformation between Cs_4_PbBr_6_ PNCs and CsPbBr_3_ PNCs

**Figure 6:**
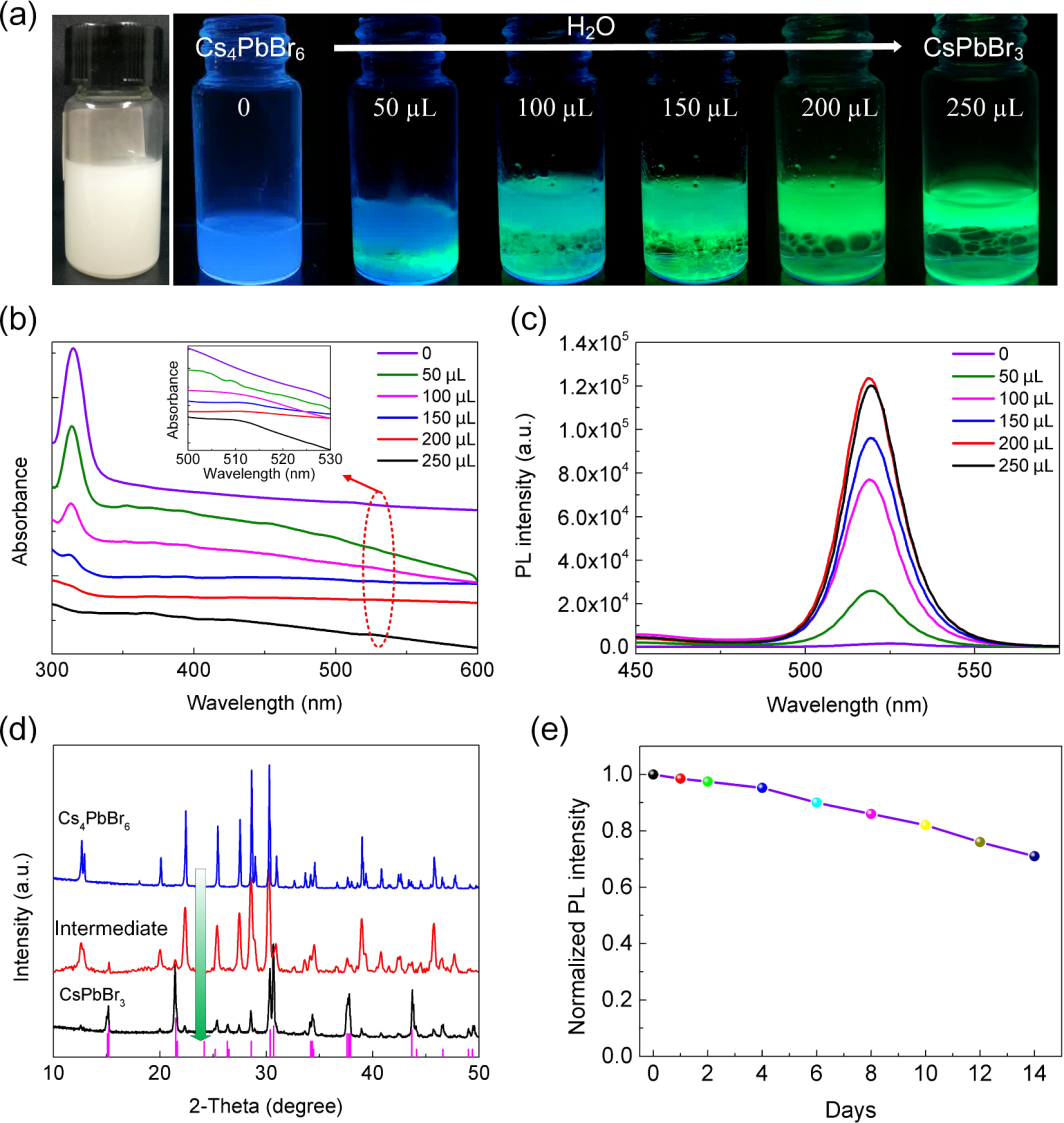
(a) The transformation process from Cs_4_PbBr_6_ PNCs to CsPbBr_3_ PNCs. The products were illuminated under a 365 nm UV light. Both (b) UV–vis absorption spectra and (c) PL spectra of products were recorded during the transformation process. (d) XRD diffraction patterns of typical products prepared by changing the amount of added water, demonstrating the transformation from rhombohedral Cs_4_PbBr_6_ PNCs to orthorhombic CsPbBr_3_ PNCs. (e) Stability of as-prepared CsPbBr_3_ PNCs in ambient environment.

After a few weeks, the prepared Cs_4_PbBr_6_ PNCs solution became milky white, indicating that untransformed CsPbBr_3_ PNCs decomposed completely. We further explored a possible reversible transformation by introducing different amounts of water. When little water was added, the color of Cs_4_PbBr_6_ solution changed from colorless to light-green rapidly (Supporting Information File 2), implying a possible structural transformation. In order to monitor the transformation process, different amounts of water were gradually dropped into a Cs_4_PbBr_6_ PNCs solution (Figure 6a). When more water was added, the solution became green-yellow.

Furthermore, the transformation process was studied by using UV–vis absorption and PL emission spectroscopy. As shown in Figure 6b, the colorless Cs_4_PbBr_6_ PNCs solution exhibits a strong first excitonic absorption peak at 315 nm. When a small amount of water was slowly added, the intensity of the first excitonic absorption peak declined gradually, indicating the decomposition of Cs_4_PbBr_6_ PNCs. Correspondingly, a weak absorption peak at 510 nm emerged. Moreover, as the amount of water was increased, the intensity of the absorption peak at about 510 nm increased steadily (inset in Figure 6b). Compared with the excitonic absorption peak of Cs_4_PbBr_6_ PNCs, the product displayed only weak absorbance. Figure 6c demonstrates the PL emission spectra of the samples during the transformation process. After the addition of small amounts of water, a PL emission peak at 518 nm appeared and gradually increased in intensity, suggesting a luminescent product was formed. XRD measurements were carried out to determine the phase of the obtained product. As shown in Figure 6d, the XRD diffraction pattern of final product is consistent with bulk orthorhombic CsPbBr_3_ (PDF card #18-0364), suggesting the formation of CsPbBr_3_ PNCs. Moreover, the PL QY of as-prepared CsPbBr_3_ PNCs was calculated to be ca. 70%. Interestingly, the CsPbBr_3_ PNCs show a high stability in ambient environment, as shown in Figure 6e. Upon the addition of a large amount of OAm and upon ultrasonication, the conversion from CsPbBr_3_ PNCs to Cs_4_PbBr_6_ PNCs was achieved (Supporting Information File 1, Figure S3) and can be repeated more than two times, similar to previous reports [25].

The addition of led to the decomposition of Cs_4_PbBr_6_ and the formation of CsPbBr_3_ PNCs, triggered by the stripping of water-soluble CsBr. During this process, the rhombohedral Cs_4_PbBr_6_ PNCs are slowly converted to orthorhombic CsPbBr_3_ PNCs (Figure 6d) and the rate of this conversion depends on the amount of water. The CsBr-stripping can be proven by the reduction of crystal size from 65 to 11.7 nm (Figure 1 and Figure 4). This is consistent with the findings reported by Wu and co-workers [20]. As the solubility of liquid paraffin or capping ligands in water is very low, further dissolution of CsPbBr_3_ PNCs is inhibited, which is similar to the effect demonstrated by Wu’s group who took advantage of the very low (only 9.5 mg/L) solubility of hexane in water [20]. The above result indicates that the CsPbBr_3_ PNCs have a higher stability than Cs_4_PbBr_6_ PNCs against water.

## Conclusion

In summary, we demonstrate the effect of small changes in the environment of capping ligands and water on the crystal structure and stoichiometry of PNCs. This study expanded our recent work of synthesizing differently shaped CsPbBr_3_ PNCs [30]. Similarly as demonstrated in our recent study, by changing the ultrasound power and radiation time, the PL emission of CsPbBr_3_ PNCs could be easily tuned. More importantly, with lower the immersion heights of the vibrating spear higher PL QY of CsPbBr_3_ PNCs were achieved. The as-prepared CsPbBr_3_ PNCs show a high PL QY of up to 85% and a considerable photostability and chemical stability. The Cs_4_PbBr_6_ PNCs are obtained via direct ultrasonication of precursors or after adding OAm in the pre-synthesized CsPbBr_3_ PNCs solution. The phase transformation of orthorhombic CsPbBr_3_ NCs to rhombohedral Cs_4_PbBr_6_ NCs is promoted by the capacity of organic ligands to dissolve PbBr_2_, and by the formation of lead oleate and the dissociation of CsPbBr_3_ PNCs. Morphology changes are mainly ascribed to the anisotropic growth of the crystals. In addition, a reverse transformation from Cs_4_PbBr_6_ PNCs to CsPbBr_3_ PNCs can be achieved by adding water to pre-synthesized Cs_4_PbBr_6_ PNCs. The developed ultrasonication assistance results in the successful control over the phase transformation of PNCs, which can find widespread application in photoelectronic devices. We anticipate that this work can be extended to prepare other halide perovskites.

## Experimental

### Chemicals

Cesium carbonate (Cs_2_CO_3_, 99%), lead bromide (PbBr_2_, 98%), liquid paraffin (90%), oleic acid (OA, 90%), oleylamine (OAm, 70%), and anhydrous toluene (99.8%) were purchased from Shanghai Aladdin Biochemical Technology Co. The chemicals used in the present work were of analytical grade and used without further purifications.

### Synthesis of CsPbBr_3_ PNCs and Cs_4_PbBr_6_ PNCs

**CsPbBr_3_ PNCs:** The PNCs were prepared via modifying the procedures reported by Tong and Rao and co-workers [29–30]. In a typical process, Cs_2_CO_3_ (0.15 mmol) and PbBr_2_ (0.30 mmol) powders were added to a mixture of 10 mL liquid paraffin (LP), 0.50 mL OA and 0.50 mL OAm. Then the reaction medium was processed by tip-sonication at a power of 120 W for 40 min. During the sonication, the colorless reaction medium gradually transformed into a yellow and then an orange-yellow solution, which suggests the formation of PNCs and demonstrates strong fluorescence emission under 365 nm UV light excitation. After completion of the reaction, unreacted precursors and excess ligands were removed by centrifugation at a speed of 3000 rpm for 10 min and then the precipitate was redispersed in 5.0 mL of toluene. Then, the colloidal solution was centrifuged at a speed of 12000 rpm for 5 min and the sediment was redispersed in toluene for further characterization.

**Cs_4_PbBr_6_ PNCs:** Cs_2_CO_3_ (0.15 mmol) and PbBr_2_ (0.30 mmol) powders were added to a mixture of 10 mL liquid paraffin, 0.50 mL OA and 3.0 mL OAm, while keeping other synthesis conditions as the same as that of CsPbBr_3_ PNCs.

### Reversible transformation from Cs_4_PbBr_6_ PNCs to CsPbBr_3_ PNCs

50–250 μL of water was added to 5.0 mL of the pre-synthesized Cs_4_PbBr_6_ PNCs solution and shaken slightly, which is a modification of the work reported by Wu and co-workers [20].

### Characterizations

The crystal surface morphology of the PNCs was characterized by transmission electron microscopy (TEM, JEM-2100F, JEOL, Japan) with an accelerating voltage of 100 kV. High-resolution TEM (HRTEM) was carried out on a JEOL JEM-2100F instrument operating at 200 kV. The crystal phases of the products were measured using an X-ray diffractometer (XRD, D8-Advance, Bruker, Germany) with a Cu Kα radiation source (λ = 0.15418 nm) at a counting rate of 2° per minute in the scanning angle (2θ) range from 5° to 50°. The surface morphology of Cs_4_PbBr_6_ PNCs was characterized by using a field-emission scanning electron microscope (SEM, Merlin). The UV–vis absorption spectra of the samples were measured using a UV–vis spectrometer (Shimadzu, Japan) over the wavelength range from 300 to 700 nm, at 1 nm intervals. The PL spectra of the PNCs were recorded using a fluorescence spectrophotometer (RF-6000, Shimadzu, Japan) using a Xe lamp as an excitation source.

## Supporting Information

File 1 Additional PL spectra, SEM image, and UV–vis absorption spectra.

File 2 Video showing the transformation from Cs_4_PbBr_6_ to CsPbBr_3_ PNCs after addition of water.
