# Supplementary material for: Ultrasonication-assisted synthesis of CsPbBr3 and Cs4PbBr6 perovskite nanocrystals and their reversible transformation
Source: Beilstein J Nanotechnol. 2019 Mar 6;10:666–76. doi: 10.3762/bjnano.10.66 (PMC6423595; doi:10.3762/bjnano.10.66)
Supplement: File 1 — Additional PL spectra, SEM image, and UV–vis absorption spectra. [file Beilstein_J_Nanotechnol-10-666-s001.pdf]

BEILSTEIN JOURNAL OF NANOTECHNOLOGY

Supporting Information

for

Ultrasonication-assisted synthesis of $CsPbBr_3$ and Cs_4PbBr_6 perovskite nanocrystals and their reversible transformation

Longshi Rao, Xinrui Ding, Xuewei Du, Guanwei Liang, Yong Tang, Kairui Tang and Jin Z. Zhang

Beilstein J. Nanotechnol. **2019,** *10,* 666–676. doi:10.3762/bjnano.10.66

Additional PL spectra, SEM image, and UV–vis absorption spectra

License and Terms: This is a supporting information file under the terms of the Creative Commons Attribution License (http://creativecommons.org/licenses/by/4.0). Please note that the reuse, redistribution and reproduction in particular requires that the authors and source are credited.
The license is subject to the *Beilstein Journal of Nanotechnology* terms and conditions: (https://www.beilstein-journals.org/bjnano)

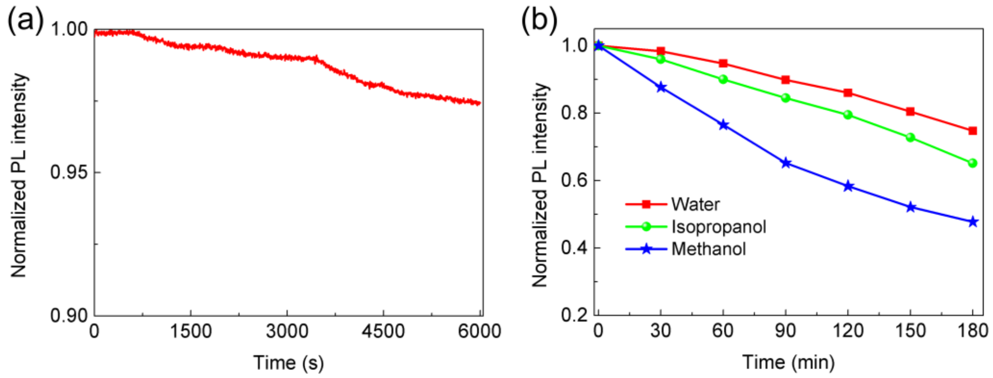

Figure S1: (a) Photostability of $CsPbBr_3$ NCs under a 365 nm UV continuous excitation. (b) The stability of $CsPbBr_3$ NCs with addition of water, isopropanol, and methanol, respectively.

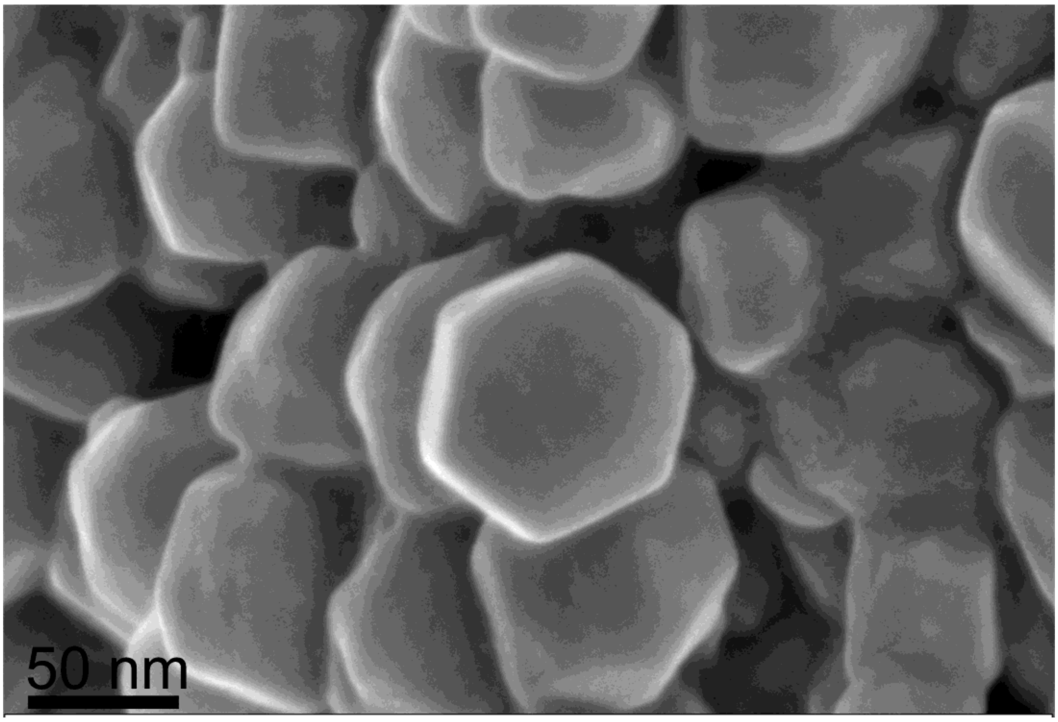

Figure S2: SEM image of Cs_4PbBr_6 NCs.

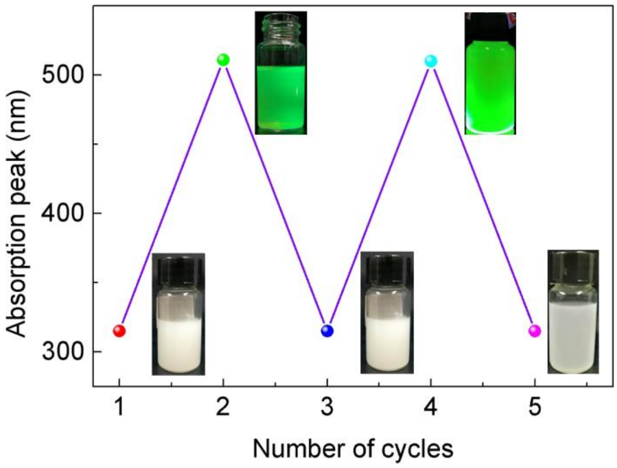

Figure S3: Conversion from $CsPbBr_3$ PNCs to Cs_4PbBr_6 PNCs. The first characteristic absorption peak of the products was recorded.
